# Electroacupuncture for postoperative pain and gastrointestinal motility after laparoscopic appendectomy (AcuLap): study protocol for a randomized controlled trial

**DOI:** 10.1186/s13063-015-0981-x

**Published:** 2015-10-14

**Authors:** Gangmi Kim

**Affiliations:** Department of Surgery, Dongguk University College of Medicine, 87 Dongdae-ro, Gyeongju-si, Gyeongsangbuk-do 780-350 Republic of Korea

**Keywords:** Electroacupuncture, Laparoscopic appendectomy, Postoperative pain, Postoperative gastrointestinal motility

## Abstract

**Background:**

Acupuncture is a widely serviced complementary medicine. Although acupuncture is suggested for managing postoperative ileus and pain, supporting evidence is weak. The AcuLap trial is designed to provide high-level evidence regarding whether or not electroacupuncture is effective in promoting gastrointestinal motility and controlling pain after laparoscopic surgery.

**Methods/design:**

This study is a prospective randomized controlled trial with a three-arm, parallel-group structure evaluating the efficacy of electroacupuncture for gastrointestinal motility and postoperative pain after laparoscopic appendectomy. Patients with appendicitis undergoing laparoscopic surgery are included and randomized into three groups: 1) electroacupuncture group, 2) sham acupuncture group, and 3) control group. Patients receive 1) acupuncture with electrostimulation or 2) fake electroacupuncture with sham device twice a day or 3) no acupuncture after laparoscopic appendectomy. The primary outcome is time to first passing flatus after operation. Secondary outcomes include postoperative pain, analgesics, nausea/vomiting, bowel motility, time to tolerable diet, complications, hospital stay, readmission rates, time to recovery, quality of life, medical costs, and protocol failure rate. Patients and hospital staff (physicians and nurses) are blinded to which group the patient is assigned, electroacupuncture or sham acupuncture. Data analysis personnel are blinded to group assignment among all three groups. Estimated sample size to detect a minimum difference of time to first flatus with 80 % power, 5 % significance, and 10 % drop rate is 29 × 3 groups = 87 patients. Analysis will be performed according to the intention-to-treat principle.

**Discussion:**

The AcuLap trial will provide evidence on the merits and/or demerits of electroacupuncture for bowel motility recovery and pain relief after laparoscopic appendectomy.

**Trial registration:**

The trial was registered in Clinical Research Information Service (CRiS), Republic of Korea (KCT0001486) on 14 May 2015.

## Background

With advances in surgery, Enhanced Recovery After Surgery (ERAS) programs enable faster recovery after surgery. The theoretical background of an ERAS program is that minimizing pain and stress in patients undergoing surgery prevents organ dysfunction and complications and consequently enhances faster recovery. The clinical significance of ERAS programs has been proven via many clinical studies. Early enteral feeding with fast bowel motility recovery, and resultant fast recovery and shortened hospital stay are advantages of ERAS programs [1–7].

The key elements of an ERAS program include 1) patient information, 2) preservation of gastrointestinal function, 3) minimization of organ dysfunction, 4) active pain control, and 5) promotion of patient autonomy [8, 9]. Many surgery centers are adopting ERAS programs and trying to enhance recovery of surgical patients by means of preserving gastrointestinal function and adequately controlling postoperative pain.

Meanwhile, we searched the literature to find other factors for recovery of surgical patients, and the literature review revealed that electroacupuncture might have positive effects on postoperative bowel motility recovery and pain control [10, 11]. Accordingly, we assumed that electroacupuncture, which is not included in existing ERAS programs, could accelerate recovery after surgery by means of postoperative pain control and faster bowel motility recovery.

This study aims to evaluate the postoperative effect of electroacupuncture on bowel motility recovery and pain relief in patients undergoing laparoscopic appendectomy.

## Methods/design

### Trial design

**Fig. 1:**
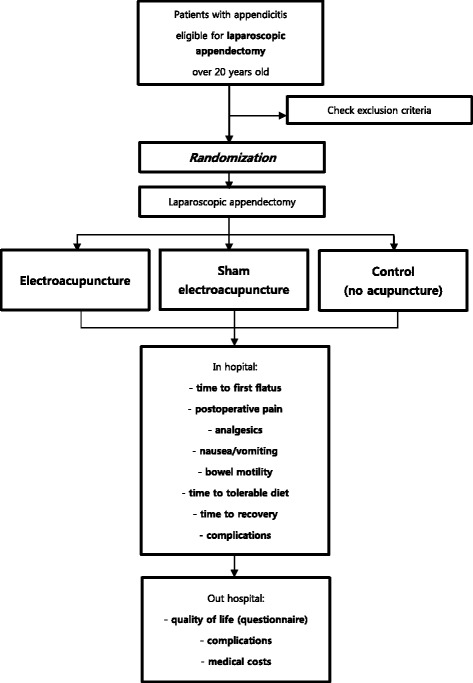
AcuLap trial flowchart

The AcuLap trial is a single-center, prospective randomized controlled trial with a three-arm, parallel-group design (Fig. 1). Data analysis will be performed according to the intention-to-treat principle.

### Methods: participants, interventions, and outcomes

Patients diagnosed with appendicitis and undergoing laparoscopic appendectomy at Dongguk University Gyeongju Hospital will be recruited.

### Eligibility criteria

#### Patient screening

1. History taking and physical examination
2. Laboratory test including CBC (complete blood count), CRP (C-reactive protein), serum chemistry, and urinalysis
3. Radiologic test including abdominal X-ray and CT (computed tomography) scan or ultrasonography.

#### Inclusion criteria

Inclusion criteria include the following:

1. Patients diagnosed with appendicitis
2. Patients over 20 years old
3. Patients undergoing laparoscopic appendectomy

#### Exclusion criteria

Exclusion criteria include the following:

1. Patients who need simultaneous combined surgery (including ileocecectomy)
2. Patients diagnosed with combined other diseases as well as appendicitis, preoperatively or intraoperatively (such as colonic diverticulitis, inflammatory bowel disease, and pelvic inflammatory disease)
3. Patients who need postoperative fasting according to preoperative evaluation or intraoperative findings (for example, panperitonitis with intraperitoneal abscess)
4. Patients under treatment for acute disease other than appendicitis
5. Patients converted to open surgery
6. Patients with cardiac pacemaker
7. Patients with allergy to or phobia of acupuncture needle or electrostimulation
8. Patients with history of syncope or seizure
9. Pregnant women or lactating women
10. Patients with history of abdominal surgery
11. Patients with American Society of Anesthesiologists (ASA) physical status classification IV
12. Patients who do not consent to clinical trial
13. Patients incapable of reading, understanding, and signing a written consent form (for example, people who are mentally retarded, blinded, illiterate, or foreigners)
14. Inmates of a prison or institution/hospital

#### Participating center/physicians

The study will be conducted in the Department of Surgery, Dongguk University Gyeongju Hospital, Korea.

Laparoscopic appendectomy will be performed by three board certified surgeons with minimum experience of 8 years.

Acupuncture will be performed by two board certified oriental medicine doctors with minimum experience of 10 years.

### Interventions

#### Study outline

Patients with appendicitis undergoing laparoscopic surgery are included and randomized into three groups: 1) electroacupuncture group, 2) sham acupuncture group, and 3) no acupuncture group. Patient allocation is performed by block randomization.

After laparoscopic appendectomy, routine postoperative care is equally given to all patients except for interventions (electroacupuncture or sham acupuncture). Clinical outcomes including postoperative pain score and bowel motility recovery are evaluated in each of the three groups during hospitalization. Quality of life and medical cost are evaluated after discharge.

#### Intervention group I: true electroacupuncture

Patients in the electroacupuncture group receive acupuncture with electrostimulation with stainless steel needles and a PG-306 electroacupuncture device after the operation. At least 2 hours after the operation, electroacupuncture is given for 30 minutes, twice a day, with a minimum interval of 4 hours between the morning session and afternoon session, starting on operation day. A maximum of four acupuncture treatments are performed during hospitalization. This acupuncture schedule is set from the literature review [12] and in consideration of the usual hospital stay after laparoscopic appendectomy.

Acupuncture is performed by Korean board certified oriental medicine doctors with 10 years of experience in acupuncture. Acupuncture sites (locations) are the following which are known to control pain and promote gastrointestinal motility: LI4 (Hapgok, Hegu), PC6 (Naegwan, Neiguan), KI6 (Johae, Zhaohai), and LR3 (Taechung, Taichong). Those acupoints are selected because they are well-known meridians for treating gastrointestinal disorders including low abdominal pain and are currently employed in our Korean oriental medical clinic.

Patients, physicians, and nurses are blinded to which intervention patients are allocated, electroacupuncture versus sham acupuncture.

#### Intervention group II: sham electroacupuncture

Patients in the sham acupuncture group receive fake electroacupuncture with a Park Sham Device (PSD). The PSD is a device designed for sham acupuncture which gives little stimulation but a fake sound. The PSD has a PG-306 electric pulse generator, just as in the true acupuncture. However, in the PSD, the internal wire inside PG-306 is cut so as not to deliver the electric pulse to patients, and the patients only hear continuous sound (fake sound) which resembles the electrostimulation.

Schedules for service and acupuncture sites are the same as in the electroacupuncture group.

Patients, physicians, and nurses are blinded to which intervention patients are allocated, electroacupuncture versus sham acupuncture.

#### Control group

Patients in the control group receive routine standard postoperative care without acupuncture.

#### Routine postoperative care for all participants

1. Routine NSAID (nonsteroidal anti-inflammatory drug) injection for postoperative pain control every 8 hours/additional injection on patient’s request
2. Start sips of water after bowel sound is checked by auscultation, unless patient complains of nausea or vomiting
3. Start soft diet after sips of water when patient is able to walk independently without nausea or vomiting
4. No patient controlled anesthesia (PCA)

### Outcomes

#### Primary outcome

The primary outcome is time to first passing flatus after operation.

#### Secondary outcomes

Secondary outcomes include postoperative pain, analgesics, nausea/vomiting, bowel motility, time to tolerable diet, complications, hospital stay, readmission rates, time to recovery, quality of life, medical costs, and protocol failure rate.

Postoperative pain will be checked regularly three times a day and will be recorded using the visual analog scale.

Intravenous analgesic injections will be counted daily.

Nausea and vomiting will be counted daily.

Bowel motility will be checked regularly three times a day, by attending surgeons. Bowel sound will be counted in a minute by auscultation.

Postoperative complications will be checked according to Accordion Severity Classification of Postoperative Complications [13].

Time to recovery is defined as time to ambulate and perform self-care by patient himself or herself.

Discharge is recommended when the patient meets ***discharge criteria*** as follows:

1. Controllable pain with analgesics
2. No nausea/vomiting
3. Passing flatus or feces
4. Toleration of soft diet
5. Mobilization (independent walking) and self-care
6. Controlled complications less than moderate by Accordion system (Table 1)

**Table 1:** Accordion severity classification of postoperative complications: expanded classification [13]

1. Mild	Requires only minor invasive procedures that can be done at the bedside such as insertion of intravenous lines, urinary catheters, and nasogastric tubes, and drainage of wound infections. Physiotherapy and the following drugs are allowed: antiemetics, antipyretics, analgesics, diuretics, electrolytes, and physiotherapy.
2. Moderate	Requires pharmacologic treatment with drugs other than those allowed for minor complications, for instance, antibiotics. Blood transfusions and total parenteral nutrition are also included.
3. Severe	Invasive procedure without general anesthesia. Requires management by an endoscopic, interventional procedure or re-operation^a^ without general anesthesia.
4. Severe	Operation under general anesthesia. Requires management by an operation under general anesthesia.
5. Severe	Organ system failure.^b^
6. Death	Postoperative death.

^a^An example would be a wound re-exploration under conscious sedation and/or local anesthetic

^b^Such complications would normally be managed in an increased acuity setting, but in some cases patients with complications of lower severity might also be admitted to an ICU

Quality of life will be assessed by SF-36 Health Survey questionnaire [14], on first outpatient clinic visit.

### Participant timeline

Patient enrollment will be done before operation. After eligibility screen, an informed consent will be obtained at outpatient clinic, emergency department, or ward. After enrollment, random allocation will be performed using blocks. The interventions (electroacupuncture or sham electroacupuncture) will be given twice a day after operation, from operation day to postoperative day 2. All clinical outcome variables will be assessed from operation day until discharge day; except for quality of life, readmission, medical costs, and protocol failure, which will be assessed on the first visit day (postoperative day 7) after discharge (Table 2).

**Table 2:** Time schedule of enrollment, interventions, and assessments

	Study period							
	Enrollment	Allocation	Post-allocation					Close-out
TIMEPOINT	-t_1_	0	t_0_	t_1_	t_2_	…	t_x_	t_x+1_
Enrollment:								
Eligibility screen	X							
Informed consent	X							
Allocation		X						
Interventions:								
Electroacupuncture			X	X	X			
Sham electroacupuncture			X	X	X			
Control								
Assessments:								
Passing flatus			X	X	X	X	X	
Postoperative pain			X	X	X	X	X	
Analgesics			X	X	X	X	X	
Nausea/vomiting			X	X	X	X	X	
Bowel motility			X	X	X	X	X	
Tolerability to diet			X	X	X	X	X	
Complications			X	X	X	X	X	
Time to recovery			X	X	X	X	X	
Fulfillment of defined discharge criteria			X	X	X	X	X	
Patient’s agreement to discharge			X	X	X	X	X	
Quality of life								X
Hospital stay								X
Readmission rate								X
Medical costs								X
Protocol failure rate								X

-t_1,_ time before operation; t_0_, operation day; t_1_-t_x_, postoperative day 1 to postoperative day *X* (= actual discharge day); t_x+1,_ the first visit day on outpatient clinic after discharge

### Sample size/recruitment

The primary endpoint is time to first passing flatus, which is defined as hours between end of operation and first flatus after operation. To calculate the sample size, a literature review was performed on acupuncture and its effect on bowel motility after laparoscopic surgery. The most similar study to this trial is that of Yu et al. [15]. They measured flatus time after laparoscopic cholecystectomy in an electric acupuncture group (n = 30), analgesia-pumper group (n = 30), and a control group (n = 30). The flatus time was shorter in the electric acupuncture group as compared with the other two groups [(14.77 ± 4.99) hours versus (18.50 ± 4.22) hours, *P* < 0.01; (14.77 ± 4.99) hours versus (18.17 ± 4.69) hours, *P* < 0.05].

For the AcuLap trial, the estimated number of participants to detect a minimum difference of time to first flatus with 80 % power, 5 % significance, and 10 % drop rate is 29 × 3 groups = 87 patients.

### Methods: assignment of interventions

#### Allocation

Allocation sequence will be generated using block randomization with StatsDirect 3 software by a randomizing person at the study center. After obtaining an informed consent from an eligible patient, the recruiter will be given a treatment code assigned to the patient from the centrally generated allocation sequence to which access is restricted for the recruiter.

#### Blinding

Two of the treatment codes are electroacupuncture and sham electroacupuncture; and the other code is control. Patients, physicians/nurses, and data analyzers are blinded between electroacupuncture and sham electroacupuncture. Only oriental medicine doctors who perform acupuncture know which treatment code corresponds to which procedure between two interventions.

The control group cannot be blinded.

### Methods: data collection, management, and analysis

#### Data collection methods

All data will be collected using the daily assessment form and will be recorded in case record form, from operation day to discharge day (Table 3). Postoperatively, on the first visit to the outpatient clinic, a quality of life questionnaire (SF-36) will be filled in by the patient.

**Table 3:** Daily assessment form: from operation day to discharge day

		Operation	POD1	POD2	POD3	POD4	POD5	…….	Discharge
Pain score (VAS)	1) Morning: ___								
	2) Mid-day: ___								
	3) Evening: ___								
IV analgesics	___/day								
Nausea/vomiting	Nausea: No/Yes(___/day) Vomiting: No/Yes(___/day)								
Bowel motility (bowel sound/minute)	1) Morning: __/min								
	2) Mid-day: __/min								
	3) Evening: __/min								
Intestinal passage	Flatus: Yes(___/day)/No								
	Stool: Yes(___/day)/No								
Tolerability to liquid	Yes/No								
Tolerability to soft diet	Yes/No								
Mobilization and self-care	Yes/No								
Postoperative complications (Accordion classification)	No/ 1/ 2/ 3/ 4/ 5								
Fulfillment of defined discharge criteria	___/total(5)								
Patient’s agreement to discharge	Yes/No								

*VAS* visual analogue scale, *POD* postoperative day

#### Statistical methods

Analysis of primary outcome:

Median of first flatus time will be analyzed by the Student’s *t*-test. *P* values less than 0.05 will be regarded as statistically significant.

Analysis of secondary outcomes:

Categorical data will be analyzed by the chi-square test or Fisher’s exact test. Continuous data will be analyzed by the unpaired *t*-test or Wilcoxon’s rank sum test. *P* values less than 0.05 will be regarded as statistically significant.

Missing data less than 5 % of the sample will be dropped. For missing data that amount to more than 5 %, analysis will be carried out with missing data imputation by the last observation carried forward and the maximum likelihood estimation method.

### Ethics

This trial is conducted in accordance with the principles of the Declaration of Helsinki and good clinical practice guidelines.

#### Research ethics approval

The independent medical ethics committee (Institutional Review Board of Dongguk University Gyeongju Hospital, Korea) has approved this trial (approval number: 110757-201410-HR-04-04). The trial was registered on 14 May 2015 at Clinical Research Information Service (CRiS), Republic of Korea (registration number: KCT0001486).

#### Consent/confidentiality

Attending surgeons will obtain written informed consent from all trial participants before inclusion. All collected data will be kept confidential at all times.

## Discussion

Garcia et al. reviewed 41 randomized controlled trials (RCTs) evaluating the efficacy of acupuncture for symptom management in patients with cancer [10]. Pain, nausea, and postoperative ileus were the most commonly studied symptoms. Among those RCTs, only one study, by Shen et al. [16], showed a positive conclusion for nausea with low risk of bias. For symptoms other than nausea, the efficacy of acupuncture remained undetermined because of the high risk of bias. Eleven RCTs on pain [17–27], 10 RCTs on nausea [24, 28–36], and 8 RCTs on ileus [17, 18, 23, 37–41] were identified as having high or unclear risk of bias and showed positive or negative conclusions for each symptom.

From the literature review, we concluded that acupuncture needs to be studied with a more sophisticated design to justify its unproven efficacy. The AcuLap trial aims to evaluate the postoperative effect of electroacupuncture on bowel motility recovery and pain relief in patients undergoing laparoscopic appendectomy. We anticipate that the trial will provide high-level evidence on the merits and/or demerits of electroacupuncture after laparoscopic surgery.

The strength of this study lies in proving the true effect of acupuncture on postoperative recovery by comparing patients receiving true acupuncture or sham acupuncture and a control group. If the true acupuncture group shows a better outcome compared to the sham group, the true effect of acupuncture can be justified on postsurgical patients. However, if the true acupuncture group fails to show a superior outcome to the sham group, even though the acupuncture group shows better recovery compared to the control group, a placebo effect of acupuncture should be considered. This is the first published protocol in English to investigate the efficacy of acupuncture on postoperative pain and ileus after laparoscopic appendectomy. We hope to share this protocol with any investigators who are interested in acupuncture and postsurgical recovery.

On the other hand, it is possible that the study may not reveal statistically significant positive or negative conclusions, because laparoscopic appendectomy is minimally invasive surgery, and most patients undergoing laparoscopic appendectomy show fast recovery after surgery. However, we hope to design our next study in other surgical procedures using the experiences from this study.

## Trial status

The trial has been open for recruitment since June 2015.

## Abbreviations

ASA: American Society of Anesthesiologists
CBC: complete blood count
CRP: C-reactive protein
CT: computed tomography
ERAS: Enhanced Recovery After Surgery
KI6: acupoint Johae, Zhaohai
LI4: acupoint Hapgok, Hegu
LR3: acupoint Taechung, Taichong
NSAID: nonsteroidal anti-inflammatory drug
PC6: acupoint Naegwan, Neiguan
PCA: patient controlled anesthesia
PG-306: an electric pulse generator used for electroacupuncture manufactured by Suzuki, Japan
PSD: Park Sham Device, a device designed for sham acupuncture
SF-36: a health survey questionnaire designed for evaluating quality of life
